# Teaching Telemedicine: The Next Frontier for Medical Educators

**DOI:** 10.2196/29099

**Published:** 2021-04-29

**Authors:** Maria Alcocer Alkureishi, Gena Lenti, Zi-Yi Choo, Jason Castaneda, George Weyer, Julie Oyler, Wei Wei Lee

**Affiliations:** 1 Department of Pediatrics University of Chicago Chicago, IL United States; 2 Pritzker School of Medicine, University of Chicago Chicago, IL United States; 3 Department of Medicine, University of Chicago Chicago, IL United States

**Keywords:** telemedicine, virtual visits, patient-centered care, graduate medical education, medical education, telehealth, virtual health, graduate students, education, COVID-19, pandemic

## Abstract

The COVID-19 pandemic has pushed telemedicine to the forefront of health care delivery, and for many clinicians, virtual visits are the new normal. Although telemedicine has allowed clinicians to safely care for patients from a distance during the current pandemic, its rapid adoption has outpaced clinician training and development of best practices. Additionally, telemedicine has pulled trainees into a new virtual education environment that finds them oftentimes physically separated from their preceptors. Medical educators are challenged with figuring out how to integrate learners into virtual workflows while teaching and providing patient-centered virtual care. In this viewpoint, we review principles of patient-centered care in the in-person setting, explore the concept of patient-centered virtual care, and advocate for the development and implementation of patient-centered telemedicine competencies. We also recommend strategies for teaching patient-centered virtual care, integrating trainees into virtual workflows, and developing telemedicine curricula for graduate medical education trainees by using our TELEMEDS framework as a model.

## Introduction

Virtual visits are “clinical interactions in health care that do not involve the patient and provider being in the same room at the same time” [1], such as visits conducted via telephone or videoconferencing [2]. At the start of the COVID-19 pandemic, virtual visits allowed clinicians to provide care to their ambulatory patients in a safe manner; however, for most clinicians, the speed at which they were forced to transition their practices to telemedicine did not allow time for thoughtful planning about the integration of patient-centered care practices and trainee education. Virtual visits continue to constitute a significant portion of outpatient care, and although guidance exists on how to make virtual visits more effective and patient-centered [2-6], we suspect many clinicians across various specialties are finding it difficult to master patient-centered virtual visit practices, all while trying to educate their students, residents, and fellows on the same topic. Furthermore, trainees and faculty may not be in the same physical space for virtual clinic sessions, which creates further challenges for integrating trainees into new workflows.

**Figure 1:**
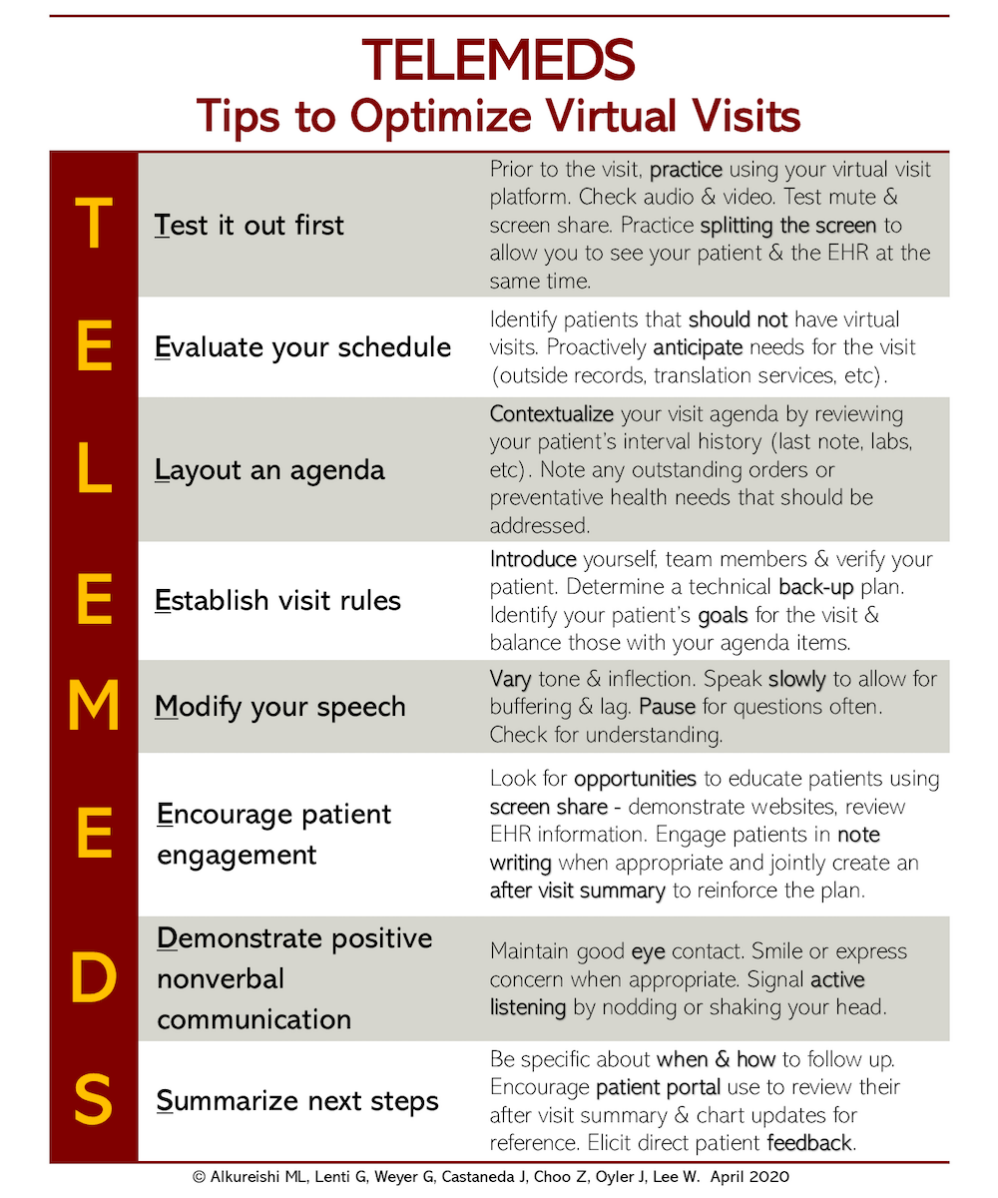
The TELEMEDS mnemonic, based on a literature review and input from key stakeholders, presents a framework for teaching patient-centered virtual practices to trainees.

Since telemedicine will likely be part of our clinical landscape in the future, clinician educators will need educational strategies to teach patient-centered virtual visit practices to trainees. Additionally, since patient-centeredness is intricately tied to care access and health equity [7], clinician educators and trainees alike must learn how to approach telemedicine from an individualized, patient-centered standpoint, understanding how it can both enhance care for some vulnerable communities [8,9] as well as ways it can widen health care disparities for others [10-13]. With this in mind, we will discuss what is known about patient-centered care, particularly as it applies to virtual visits. We will propose strategies for teaching patient-centered virtual practices to trainees with the guidance of the framework “TELEMEDS,” which is based on a literature review and input from key stakeholders, including trainees and practicing clinicians (Figure 1). Although some of the tips we share in this paper are specific to video visits and the added benefit of connecting visually across a screen, many of our strategies (eg, reviewing a virtual clinic schedule and verbal communication tips) also apply to telephone visits, so we will use the term “virtual visit” to apply broadly to both scenarios. Finally, we will discuss how best to integrate trainees into virtual clinic workflows.

Our recommendations provide practical tips for incorporating patient-centered telemedicine into clinical training; however, more work is needed to refine and implement these strategies. Thus, we recognize the need to develop telemedicine curricula for senior clinicians and trainees alike. We call on the medical education community to prioritize the development, equitable implementation, and study of evidence-based telemedicine training and the meaningful evaluation of trainees with regard to these skills.

## What We Know About Patient-Centered Care and Telemedicine

Patient-centered care is defined as “providing care that is respectful of and responsive to individual patient preferences, needs and values and ensuring that patient values guide all clinical decisions” [14]. Prior studies have shown patient-centered care in the in-person setting is associated with higher patient satisfaction and positive health outcomes [15,16]. As the patient-centered medical home [17] extends into a virtual space, the same guiding principles of patient-centered care are still possible, if not more so. In fact, simply providing virtual visit options may allow patients to access care more easily, improve communication with their care team, and give patients more control over where and how they choose to interact with the health care system—all important and fundamental tenets of providing the right care, at the right time, in the right place [18].

Additional studies have demonstrated several benefits of virtual visits, including ease of use, low cost, ability to improve patient-provider communication, decreased travel time, increased access to care for patients, and high patient satisfaction [19-21]. Despite these benefits, telemedicine may risk further fragmentation of care if not implemented correctly [22]. In particular, it raises issues related to equitable care delivery and concerns of exacerbating the digital divide, where access to the technology required for telehealth differs along sociodemographic lines [10-12]. Further, the virtual nature of telemedicine has the potential to hinder patient-provider communication; for example, in one study where patients expressed concerns about errors in their care due to the lack of physical exam, they reported feeling less involved during the visit and had difficulty finding opportunities to speak [23]. Other studies have summarized further communication drawbacks, including lack of physical touch, difficulty building rapport, and decreased ability to recognize subtle nonverbal cues and expressions [2,24].

Although we are still discovering barriers and solutions to patient-provider communication through the lens of this new technology, we can look to recent history for cues on how to overcome challenges in an increasingly tech-centric world. For instance, as electronic health records (EHRs) became the norm across institutions, studies found that providers spent more than half of their time in a patient encounter navigating the EHR system, which resulted in a struggle for providers to give adequate time to direct patient care [25]. Another study on patient perceptions of EHR use found that patients expressed concern that their physicians were more focused on the computer than on them during in-person clinic visits [26]. However, over the course of time, providers found ways to utilize the EHR to improve patient-doctor communication, to engage patients visually, and to actively promote discussion, education, and shared decision-making [25,27].

Some more recent work has helped elucidate how the core principles of patient-centered care can be applied to telemedicine. In the midst of the COVID-19 pandemic, some institutions developed checklists or principles to guide clinicians on how to carry in-person patient-centered communication into the virtual world [5,6]. Others have recommended helping patients understand their role in telemedicine communication, emphasizing the importance of preparing for and engaging in virtual visits [2]. The Association of American Medical Colleges (AAMC) has also released a report on telehealth competencies for trainees and providers across the continuum [28]. Although all these guidelines provide a base for improving patient-provider communication in the virtual setting, more evidence is needed to ascertain how these guidelines impact patients’ perceptions of their care as well as their health outcomes. Additional guidance for medical educators is also needed on how to teach these emerging “best-practices'' and competencies to trainees, how to meaningfully integrate trainees into virtual clinic workflows, and how to provide feedback on patient-centered virtual communication.

## Teaching Patient-Centered Telemedicine

Preparing for a virtual visit clinic day with trainees necessitates deliberate planning on the part of both the supervising clinician and the trainee. For virtual sessions, trainees are still expected to review their schedule, chart review, and ensure adequate follow-up for patients, all while considering the limitations of the virtual setting. Supervising clinicians should teach trainees how each of these tasks looks different in the virtual setting and coach them on how to troubleshoot technological and communication issues before they arise (Figure 1) [3,4,6]. Additionally, preceptors should pursue opportunities to teach learners how to assess which patients are *appropriate* for video or phone visits and which situations may be more suited for an in-person visit [6,24]. Supervising attendings should focus on virtual visit communication skills, efficient utilization of the visit platform, setting expectations for the visit with patients, the importance of body language and speech [3,4,6], and strategies to engage patients by using video tools such as “screen share” (Figure 1).

It is also critical to train learners on how to leverage telemedicine to do things we cannot do in the in-person clinic setting. For example, the ability to have a family member join in from a separate location for a virtual visit with their elderly parent may add critical information that would not have been obtainable otherwise [29]. Similarly, information can be gleaned by using video as an opportunity to assess relevant parts of a patient’s home environment in a way that is akin to the traditional and time-honored home-visit. In this way, video visits can be used to identify potential fall risks in a patient’s home, accurately review how patients organize and take their medications [29], or to identify safety hazards present in the homes of pediatric patients. Virtual visits can also be used to augment in-person care to allow for touchpoints between clinic visits; for example, to assess medication tolerance or symptom relief or for follow-up educational sessions that may not require a full physical exam or assessment.

Finally, it is important to foster trainee awareness of patient-related telemedicine *challenges* and to present those from the perspective of health equity and access to care. As medical educators, we must not only look for ways to educate our learners on the factors that contribute to the creation of a digital divide, but we must also proactively cultivate opportunities for trainees to become involved in advocacy and quality improvement efforts to address these barriers head-on.

## Embedding Trainees into Virtual Clinic Workflows

Integrating trainees into telehealth experiences not only provides opportunities for experiential learning and professional identity development but also contributes to improved patient health and extended capabilities of health care teams [30]. Therefore, thinking critically about the design of a virtual clinic workflow is crucial to ensuring successful clinical encounters and a supportive learning environment.

Unlike in-person clinic days where communication can be done face-to-face, virtual clinic days require clear expectations for how and when trainees should connect with patients, as well as a direct line of communication with their faculty preceptors so that they are quickly and easily accessible when needed. When multiple trainees (eg, medical student, resident, and fellow) are involved in a visit, each should have a specific role and understand how to quickly communicate with their supervisor if a need arises. Coordinating such a *dance* takes effort and skill, but with practice, it can become a meaningful care experience not just for trainees but for patients as well.

Although some clinicians may choose to communicate with trainees using nonvisual methods (eg, phone calls and text messaging) for simple questions throughout a virtual visit session, conducting an in-person or videoconference pre- and post-visit huddle can provide the added benefit of connecting in a more personal way and allows educators to read their trainees’ verbal and non-verbal cues. Additionally, post-visit sessions provide opportunities for trainees to receive feedback on their patient-centered virtual visit skills as well as for the supervising clinician to receive feedback on their workflow, communication, and patient teaching in addition to a review of their documentation using the screen share function.

In the process of workflow development, it is important to note that no workflow is perfect or universal; workflows may change as we begin to better understand how various setups impact patient-centered care. For example, if multiple trainees are involved in the same call with one patient, this may enhance education, but it may be overwhelming for the patient. This example underscores the importance of setting expectations with patients at the start of a visit and obtaining feedback at its conclusion, which will allow individual clinicians to make important and necessary changes to their workflows over time. A virtual clinic workflow may also differ across providers and institutions, depending on the needs of each organization and the infrastructure of the virtual visit platform used. Knowledge of the benefits and limitations of the technology one has access to is inherent to developing workflows for individual educators. At the institutional level, organizations should strive to integrate Health Insurance Portability and Accountability Act (HIPAA)-compliant platforms that support *various* workflows and consider trainee education along with platform selection. Furthermore, organizational buy-in is needed to integrate *time* for trainee education, debrief, and feedback sessions within a virtual clinic schedule and for observation and assessment during the continuum of their training.

## Establishing Telemedicine Curricula for Graduate Medical Education

**Figure 2:**
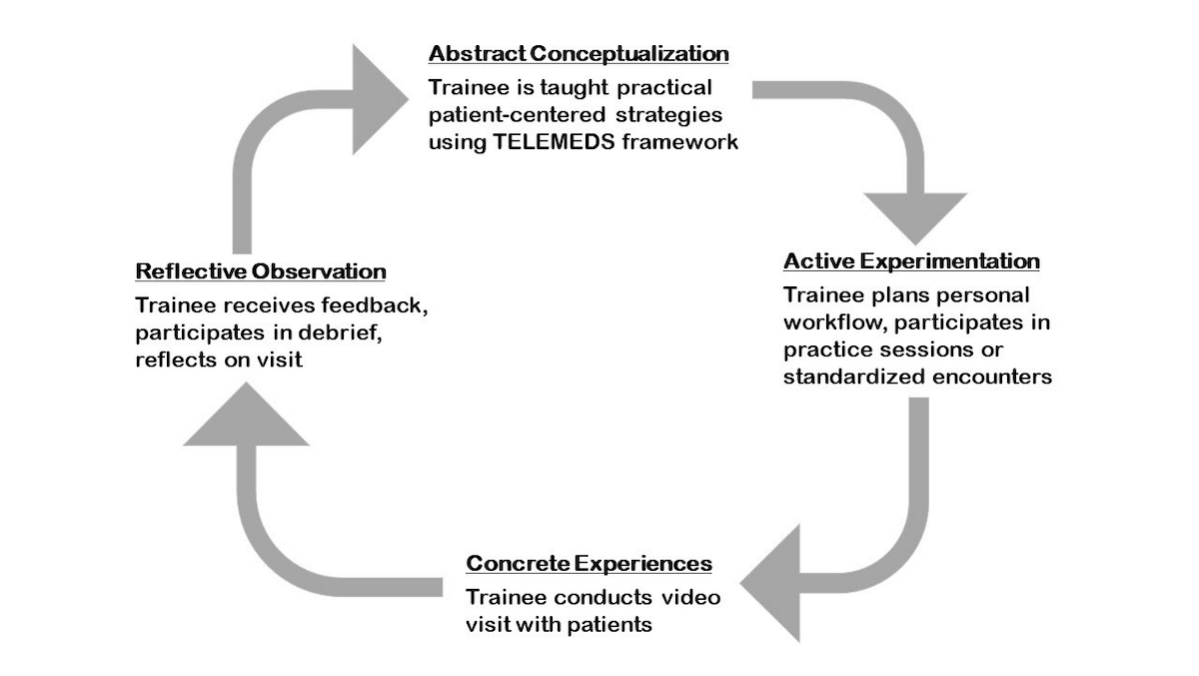
Kolb’s Experiential Learning Cycle [27]. Four stages to promote effective learning of patient-centered virtual visit practices.

Given the limited use of telemedicine prior to the COVID-19 pandemic, it is unlikely that many current trainees have received formal telemedicine training prior to or during residency. Moving forward, medical school, residency, and fellowship programs should develop purposeful telemedicine curricula for the trainees by considering the proposed AAMC telemedicine competencies and by using the aforementioned strategies and Kolb’s Experiential Learning Cycle [31], a four-stage learning theory to promote effective learning (Figure 2).

Applying Kolb’s Experiential Learning Cycle to teaching patient-centered virtual communication, the trainee should first be introduced to the TELEMEDS framework to better understand practical, patient-centered virtual communication skills (ie, abstract conceptualization). Medical educators should then provide arenas (eg, standardized encounters or virtual visit practice sessions) that reinforce the TELEMEDS concepts (ie, active experimentation) to be used when trainees conduct virtual visits with patients (ie, concrete experiences). Ideally, supervising attendings should provide real-time feedback for trainees on directly observed behaviors in order to encourage continued reflection and skill development (ie, reflective observation).

Other effective strategies for teaching patient-centered telemedicine may rely on competency-based medical education (CBME), focusing on measuring goal-oriented outcomes for learners, such as mastering the technology, performing a comprehensive video-based physical exam, and understanding professionalism in telemedicine [32]. Finally, educators should seek opportunities to serve as role models for trainees, as well as foster and nurture trainee involvement in advocacy and quality improvement efforts to improve health care access and telehealth equity for patients.

Thus, medical educators should strive to develop formal tools to guide this feedback, standardize assessment among learners, and assess how proficiency in these competencies affects patient outcomes.

## Conclusions

Virtual visits will likely be a part of our clinical world moving forward. As medical educators adjust to this new form of care delivery, it is important to take a proactive approach to educate trainees on patient-centered telemedicine practices and integrate trainees into new, thoughtful, and deliberate workflows. It is important to note that future curricula for trainees will likely parallel that for preceptors, as many faculty members may not have received prior training, and some may not yet have attained proficiency in the skills of patient-centered virtual communication or teaching telemedicine best-practices. As such, faculty development will play a large role in this process. The TELEMEDS framework can be used by senior clinicians to provide structure and meaningful feedback to trainees to improve their virtual visit skills. Although further study on virtual visit communication skills is needed, our strategies provide important initial guidance for medical educators on how to promote meaningful, patient-centered virtual care.

## Abbreviations

AAMC: Association of American Medical Colleges
CBME: competency-based medical education
EHR: electronic health record
HIPAA: Health Insurance Portability and Accountability Act
